# Unveiling abietic Acid’s therapeutic potential: a narrative review on structure-activity relationship, pharmacological properties, pharmacokinetics, and toxicological considerations

**DOI:** 10.3389/fphar.2025.1738572

**Published:** 2026-01-09

**Authors:** Wanqing Ren, Tongzheng Liu, Jianlin Wu

**Affiliations:** College of Traditional Chinese Medicine, Shandong University of Traditional Chinese Medicine, Jinan, China

**Keywords:** abietic acid, pharmacokinetics, pharmacological properties, structure-activity relationship, toxicology

## Abstract

Abietic acid (AA) is a carboxylic acid and a tricyclic diterpenoid that is found in a variety of coniferous resins. Its promising natural properties have renewed interest in this substance that has been used medicinally for centuries for a range of indications including wounds, inflammation and infections. Extensive preclinical evidence over the past decade also supports its therapeutic properties. This review discusses the structure-activity relationship, pharmacological actions, pharmacokinetics and toxicology of AA. The unique molecular structure of AA, which supports a phenanthrene-like structure, a conjugated diene and carboxylic acid pharmacophore, rationalizes aspects of its wide biological effects. Preclinical research supports potentially significant anti-tumor effects in model systems through ferroptosis and cell cycle arrest, prominent anti-inflammatory activity via COX-2 inhibition and PPARα/γ activation, broad-spectrum antimicrobial activity with antibiofilm effects and hepatoprotective effects via Nrf2/HO-1. Nevertheless, clinical translation is faced with low oral bioavailability, poor aqueous solubility, and high first pass metabolism. New delivery systems such as nanoparticle formulations may hold promise in overcoming these challenges. Although its toxicological profile appears favorable, thorough pharmacokinetic studies and well-designed clinical trials are necessary. This narrative review highlights the novelty of consolidating scattered preclinical data on AA, a lesser-known natural compound in pharmacology, to increase awareness of its multifaceted therapeutic potential. Its utility lies in guiding future research toward optimized derivatives and formulations, potentially bridging traditional medicine with modern therapeutics for conditions like cancer, inflammation, and infections, while identifying key gaps for interdisciplinary efforts.

## 1 Introduction

**FIGURE 1:**
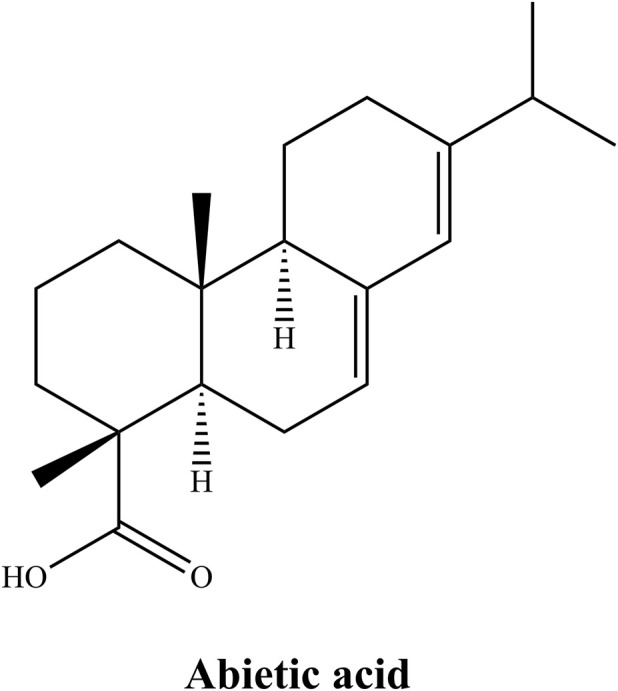
Chemical structure of AA.

Abietic acid (AA) is a tricyclic diterpenoid carboxylic acid found primarily in coniferous resins of trees such as spruce and pine (Shuai et al., 2021; Wu et al., 2020) (Figure 1). The complex molecular structure of AA includes a fused phenanthrene ring system, conjugated double bonds, and a carboxyl group situated on C-18 (Sultanova et al., 2024). This unique chemical structure provides AA with several biological activities and interactions. The therapeutic use of resins containing AA has been historically documented; Indigenous societies located in North America and Eurasia have traditionally utilized these natural products for ailments such as wounds, inflammation, and microbial infections (Ahmad et al., 2024; Ramnath et al., 2016). Pioneering records in traditional Chinese medicine also describe the use of pine resin products for treating cancer (Wen et al., 2019). More contemporary studies conducted in pharmacology have supported these uses, and it has since been reported that AA possesses considerable anti-tumor (Ahmad et al., 2024), anti-inflammatory (Sierra et al., 2022), antimicrobial (McCadden et al., 2025), and hepatoprotective effects (Jung et al., 2022). These properties have also demonstrated selectivity toward malignant cells with low toxicity toward normal cells at concentrations relevant for therapeutic use, supporting the claim of its potential to serve as a viable chemotherapy agent (An et al., 2023; Ozsen et al., 2017).

The development of extraction and purification techniques—especially supercritical fluid chromatography and biocatalytic derivatization—has allowed for high-purity AA and synthetic derivatives, and enabled careful structure–activity relationship (SAR) studies (Falev et al., 2022; Feitosa et al., 2025). SAR research shows that AA bioactivity is closely linked to its tricyclic scaffold, functional groups (e.g., hydroxyls), and stereochemistry (Jeon et al., 2015; Li et al., 2020). Even minor structural modifications, such as esterification of the carboxyl group or hydroxylation of the core scaffold at specific positions, can significantly improve bioavailability and target selectivity (Mitsukura et al., 2005). As an example, methyl abietate derivatives demonstrate superior aqueous solubility and enhanced anticancer activity compared to the parent compound (Li et al., 2019). These observations are consistent with the trend in medicinal chemistry to use natural products scaffolds when designing new therapeutics, especially to achieve increased efficacy and decreased toxicity.

Although AA has several promising properties, its translational development is hindered by the lack of clinical evidence, incomplete understanding of its molecular mechanisms, and insufficient evaluation of synthetic analogs developed to improve pharmacokinetics and target engagement. Addressing these gaps will require an interdisciplinary effort that includes the integration of phytochemistry, pharmacology, and toxicology (Qiu et al., 2020).

The aim of this narrative review is to consolidate the current state of knowledge on AA by providing insights into its SAR, pharmacological properties, pharmacokinetics, and toxicological considerations, with the goal of highlighting its therapeutic potential and identifying priority areas for future research.

### 1.1 Methodology

The narrative review was performed through electronic database searches (PubMed, Scopus, Web of Science, Google Scholar, and Sci-Finder), with the date range of publication from the beginning of the database to December 2025. The search terms used included the combinations of the Project TAG with the search triggers of Structure Activity Relationship (SAR), pharmacological properties, anti-tumor, anti-inflammatory, antimicrobial, hepatoprotective, pharmacokinetics, toxicology, and pre-clinical studies (which included both *in vitro* studies and *in vivo* studies) via the use of “abietic acid” as the trigger word. All languages were reviewed; however, the only articles that were reviewed were in the English language. The inclusion criteria were research articles, reviews, and studies that described the chemical structures, biological activity, pharmacokinetics, and toxicity of AA studied in preclinical models. The exclusion criteria for this review were non-peer-reviewed literature, studies on compounds not related to AA, and studies that did not provide enough data to be considered as studies. Approximately sixty relevant articles were selected for inclusion based on relevance and quality; however, because the authors wanted to focus on publishing up-to-date material, they prioritized those articles published after 2015. The authors did not conduct a formal meta-analysis, as this research narrative is a narrative review, but they synthesized the information from the included studies by thematic analysis of the information. The reference lists for the studies that met the inclusion criteria were also evaluated for additional studies.

## 2 SAR

In general, a pharmacological profile is strongly associated with a compound’s unique molecular structure (Bai et al., 2025; Zheng et al., 2017). AA’s tricyclic abietane framework, carboxylic acid functional group, and hydrophobic moieties allow for a range of biological interactions that promote its anti-inflammatory, anticancer, antioxidant, and antimicrobial activity (Hashimoto et al., 2023; Luo et al., 2025). For this reason, a detailed examination of the SAR of AA is warranted for targeted drug design and the creation of potential new derivatives. This section will identify the roles of specific structural features in the bioactivities of AA, highlighting recent studies and key insights.

### 2.1 Structural backbone: the tricyclic diterpenoid framework

The basis for AA activity is the tricyclic phenanthrene-like scaffold which has two angular methyl groups on the C-16 and C-17 positions (Gaborova et al., 2021; Zentar et al., 2022). The overall rigid, planar structure permits π-π stacking interactions within hydrophobic binding pockets of specific proteins that increase specificity and binding affinity (Rahaman et al., 2024). The chair-boat conformation of the cyclohexane ring allows for adaptable binding to distinct biological entities. The extended conjugated diene structure (i.e. Δ7,8 and Δ13,14) allow for electron delocalization that increases free radical scavenging potential and increases capacity for generating reactive oxygen species (ROS), a characteristic that is plausible in cancer cell ferroptosis induction (Xu et al., 2023).

The scaffold’s natural hydrophobicity allows selective partitioning into lipid bilayers, which can then modulate membrane-associated signaling pathways. This property is essential for AA to accumulate in cellular membranes and impact processes like inflammation and apoptosis. Structural analogs of AA, such as dehydroabietic acid, which share the same core but have distinct oxidation states, have demonstrated similar or greater antimicrobial and anti-inflammatory properties compared to AA, demonstrating the diverse applications of the scaffold. Of note, the planar scaffold may also inhibit AA’s solubility and bioavailability leading to challenges in pharmaceutical development. Optimizations, such as nanoparticle-based AA delivery, have been utilized to enhance AA delivery and efficacy, providing an understanding of the tradeoff between scaffold rigidity and drug formulation/development (Hao et al., 2022).

### 2.2 Carboxylic acid group: a key pharmacophore

The carboxylic acid group at C-18 is a vital pharmacophore that dictate AA’s electronic properties, as well as its solubility and interactions related to molecular activity. In its ionized form as a carboxylate, it is responsible for pH-dependent interactions with inflammatory mediators, such as inhibiting the activation of NF-κB (nuclear factor kappa-light-chain-enhancer of activated B cells) and reducing the production of TNF-α (tumor necrosis factor-alpha) and IL-1β (interleukin-1 beta) (An et al., 2023). Esterifying or removing this group eliminates anti-inflammatory effects, indicating its importance in target binding.

Additionally, the carboxylic acid group is instrumental in AA’s properties as an antioxidant and a regulator of ferroptosis. The carboxylate group provides the ability for free radical scavenging via proton-coupled electron transfer, and the ability to chelate Fe^2+^ minimizes labile iron pools and limits processes such as lipid oxidation, ultimately reducing the formation of 4-hydroxynonenal (Shi et al., 2014; Zhang et al., 2023). Furthermore, the carboxylic acid group promotes enhanced binding of AA to discrete enzymes such as GPX4 (glutathione peroxidase 4) through hydrogen bonding at the active site (where there is a selenocystine), allowing for enhanced anti-ferroptotic effects observed in AA, compared to other non-acidic diterpenoids (Shi et al., 2014; Zhang et al., 2023).

In terms of pharmacokinetic properties and microenvironment, the ionization of this functional group enables AA to cross biological barriers, while simultaneously permitting enough hydrophilicity for aqueous reactivity (Villalain, 1997). Structural studies demonstrate that the planar arrangement between the carboxyl and adjacent methyl positions yields a stereo electronic environment that is best suited for “dual” interactions with biological targets, in both hydrophilic and hydrophobic environments (Labrecque and Fuglestad, 2021; Zhang et al., 2023). This dual functionality underscores the carboxylic acid as a multifunctional moiety essential for AA’s pleiotropic actions.

### 2.3 Hydrophobic groups: enhancing membrane affinity and target binding

The hydrophobic properties of AA, which include the isopropyl group (C-13) and methyl groups (C-10 and C-16), are important determinants for AA’s membrane permeability and binding efficiency to target proteins. The β-orientated isopropyl group interacts with the hydrophobic pocket of proteins, such as IKKβ (IκB kinase beta), which stabilizes the binding complex through van der Waals forces and enhances a broad range of effects involving anti-inflammatory signal transduction (Jung et al., 2022; Kang et al., 2018). The C-10 methyl group also helps conformationally stabilize the A ring and protects the carboxylic acid functional group from a nucleophilic attack, reducing the consumption of AA’s reactive carbons (Rafferty et al., 2013).

Notably, the hydrophobic “clamp” formed by the C-10 methyl and C-13 isopropyl groups provides a two-fold increase in the activation of PPARγ (peroxisome proliferator-activated receptor gamma) by simultaneously engaging the Leu330 and Phe363 residues of the ligand-binding domain (Fernandez, 2014). Additionally, the conjugated double bonds (Δ7,8 and Δ13,14) promote π-π stacking with aromatic amino acids contributing approximately 15% of the total binding energy (Gonzalez et al., 2009; Huang et al., 2017). Additionally, the Δ13,14 double bond provides stable planarity that is optimal for recognition by the target protein, which is demonstrated by the loss of activity by saturation analogs (Gonzalez et al., 2009).

The hydrophobic residues also facilitate the incorporation of AA into lipid membranes modulating AA’s pharmacological effects on membrane-bound receptors and enzymes. At the same time, excessive hydrophobicity may decrease aqueous solubility, which may require structural trade-offs. Semisynthetic modifications, such as introducing polar substituents to the isopropyl group, have yielded derivatives with improved bioavailability and retained activity, illustrating the potential for rational optimization.

### 2.4 Stereochemistry: the role of chirality in target specificity

The stereochemical configuration of AA determines its 3D orientation and target selectivity (Tapia et al., 2014). The 4R chiral center and position of the C-4 methyl group enables stabilization of the C-ring in a half-chair conformation to maximize the hydrogen bonding network of the carboxylic acid with Ser177 residue in PPARγ (Fernandez, 2014; Masnyk et al., 2018; Thummuri et al., 2018). Molecular docking studies support that the R-configuration at C-9 establishes optimal hydrophobic packing into the PPARγ ligand-binding domain and results in transcriptional activation (Jeon et al., 2015).

The chiral crown conformation of AA’s tricyclic system is even capable of differential engagement with distinct targets: the concave β-surface engages with the helix 3/β-sheet region of the PPARα (peroxisome proliferator-activated receptor alpha) target, while the convex α-face of AA engages with TRPV1 channels via van der Waals forces (Masnyk et al., 2018; Oyama et al., 2021; Wang et al., 2011). This stereospecificity is important in selectivity for PPARγ over LXRβ (liver X receptor beta) and for minimizing off-target effects (Ohashi et al., 2010; Yue et al., 2005). For example, simple epimerization at C-4a can completely obliterate NF-κB inhibition, demonstrating the sensitivity of bioactivity to stereochemistry change.

**FIGURE 2:**
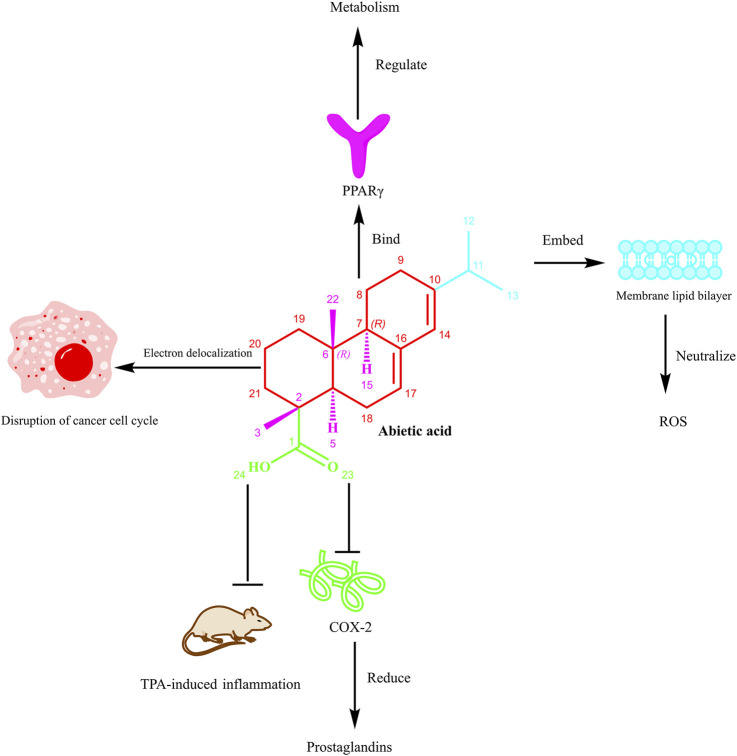
The SAR of AA. Note: Figure 1 illustrates the multifaceted biological actions of AA. The tricyclic scaffold, carboxylic acid group at C-18, hydrophobic moieties (e.g., isopropyl at C-13 and methyl groups at C-10, C-16), and stereochemistry (e.g., 4R chiral center) collectively contribute to its activities. Specifically, AA can bind to PPARγ (peroxisome proliferator-activated receptor gamma) to regulate metabolism. Its structural moieties, including the embedded segment within the membrane lipid bilayer, contribute to neutralizing ROS (reactive oxygen species). The electron delocalization feature of AA disrupts the cancer cell cycle. Moreover, it inhibits COX-2 (cyclooxygenase-2), thereby reducing prostaglandin production and suppressing TPA (12-O-tetradecanoylphorbol-13-acetate)-induced inflammation *in vivo*. This figure emphasizes how modifications to these features can enhance bioavailability, target selectivity, and therapeutic efficacy, guiding derivative design for improved pharmacological profiles.

Asymmetric synthetic methodologies that produce stereoisomers for SAR have advanced to the point where we can now show that minor chiral changes can result in dramatic alterations in potency and selectivity. Future advances to utilize the stereochemical features of AA may lead to highly specific modulators of metabolic and inflammatory pathways. The brief SAR of AA is illustrated in Figure 2.

## 3 Pharmacological properties

### 3.1 Anti-tumor activity

Studies show that AA exhibits cytotoxicity on a wide variety of cancer cells but has little effect on normal cells, suggesting a favorable therapeutic window. The mechanisms of its anticancer effect are complex and vary among cancer cell types, including induction of programmed cell death, cell cycle inhibition, anti-metastatic activity, and modulation of various oncogenic signaling pathways.

One interesting observation is the ability of AA to induce ferroptosis, which is a form of programmed cell death that is iron-dependent, in bladder cancer cells. Xu et al. reported that AA selectively reduced the viability of bladder cancer cells (e.g., by 50%–70% in T24 and 5637 cell lines at 10–20 μM) but did not have an effect on the normal urothelial cells (Xu et al., 2023). This effect was mediated by upregulation of heme oxygenase-1 (HO-1), followed by downregulation of glutathione peroxidase 4 (GPX4) and accumulation of ROS, lipid peroxidation, and iron overload, which are all biomarkers of ferroptosis. The criticality of this pathway was shown when forced expression of GPX4 or pharmacological inhibition of HO-1 attenuated ferroptosis from AA. AA also acted synergistically with classical chemotherapeutics such as cisplatin and gemcitabine and reduced *in vivo* tumor growth in xenograft mouse models, making it a promising candidate for bladder cancer treatment (Xu et al., 2023).

In non-small cell lung cancer (NSCLC), AA inhibited cell proliferation and colony formation by blocking the IKKβ/NF-κB signaling pathway. Liu et al. showed that AA bound directly to IKKβ, leading to decreased phosphorylation of IKKβ and the inhibition of NF-κB nuclear translocation (Liu et al., 2019). NF-κB is a key modulator of genes regulating apoptosis and cell growth, thus, the intervention of the IKKβ/NF-κB pathway resulted in apoptosis as indicated by G0/G1 phase cell cycle arrest, decreased Bcl-2, and PARP (poly (ADP-ribose) polymerase) cleavage. Furthermore, overexpression of IKKβ reversed the tumor inhibitory effects of AA, showing that inhibition of IKKβ onset AA cytotoxicity. Computational molecular dynamics simulations suggested that hydrophobic interactions, among other factors, drive the binding interaction of AA and IKKβ (Liu et al., 2019).

AA’s antimetastatic capability further enhances its wide-ranging antitumor activity. For example, AA inhibited cell migration, invasion, and motility *in vitro* in melanoma even without negatively affecting cell viability at the antimetastatic levels (Hsieh et al., 2015). Inhibition of migration, invasion, and motility was consistent with decreased activities of the matrix metalloproteinase-2 (MMP-2) and the urokinase-type plasminogen activator; two highly involved enzymes associated with degradation of the extracellular matrix and promoting cancer dissemination. Furthermore, inhibition of these proteases coincided with the inhibition of the PI3K/Akt (phosphatidylinositol 3-kinase/protein kinase B) signaling pathway and decreased DNA binding activity of the transcription factor AP-1 (activator protein-1). Furthermore, AA decreased lung metastasis of B16F10 melanoma cells by approximately 92.8% *in vivo* when administered orally, demonstrating the potential to inhibit cancer dissemination (Hsieh et al., 2015).

Similar mechanisms have been observed in other cancer types. For instance, in nasopharyngeal carcinoma, AA also induced G2/M phase cell cycle arrest and mitochondrial-mediated apoptosis ascribed to the generation of ROS, loss of mitochondrial membrane potential and activation of caspases-3 and -9 (Wen et al., 2019). AA also significantly inhibited migration and invasion of NPC cells (e.g., reducing invasion by up to 60% at 20 μM) and inhibited the PI3K/Akt/mTOR (mammalian target of rapamycin) pathway which is a key regulator of cell growth and survival. In addition, AA showed chemopreventive activity in a mouse model of skin cancer in which AA inhibited DMBA (7,12-dimethylbenz [a]anthracene) and UVC-induced tumor formation likely through downregulation of MMPs (matrix metalloproteinases) and cytokeratin (Yang and Tian, 2017).

**FIGURE 3:**
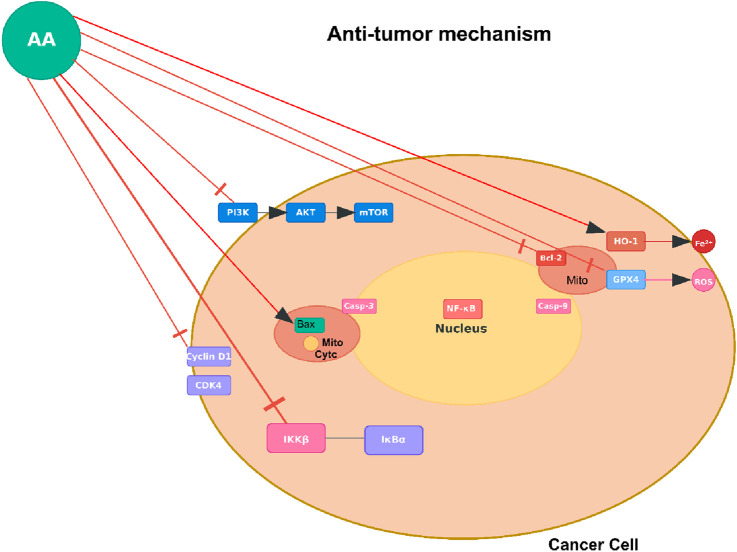
Anti-tumor mechanism of AA. Note: Figure 3 shows AA targeting oncogenic pathways (e.g., RTK-AKT-mTOR [receptor tyrosine kinase - protein kinase B- mammalian target of rapamycin]) and regulators (HDAC [histone deacetylase], GPX4 [glutathione peroxidase 4]) in cancer cells to inhibit proliferation or induce death. This represents one of several reported mechanisms, observed in tumor types such as bladder cancer (via ferroptosis induction), non-small cell lung cancer (NSCLC; via IKKβ/NF-κB inhibition), melanoma (via anti-metastatic effects on MMP-2 [matrix metalloproteinase-2] and PI3K/Akt), nasopharyngeal carcinoma (NPC; via G2/M arrest and PI3K/Akt/mTOR), and skin cancer (via MMP downregulation).

The evidence for the antitumor effects of AA is robust and a result of a complex interplay of mechanisms including induction of ferroptosis, apoptosis, cell cycle arrest, inhibition of key signaling pathways such as IKKβ/NF-κB and the PI3K/Akt/mTOR pathway, and inhibition of metastasis. The preferential effects on cancer cells and the ability to synergize with existing chemotherapeutics add to the therapeutic appeal of AA. Nonetheless, while preclinical data are optimistic, more research is needed to fully understand its pharmacokinetics, assess *in vivo* efficacy in various cancer models and assess potential toxicities before finally translating AA to the clinic (Figure 3).

### 3.2 Anti-inflammatory activity

Numerous preclinical investigations using diverse inflammatory models have confirmed the anti-inflammatory effects of AA. The initial study showed that AA concentration-dependently inhibited protein denaturation and protected *in vitro* human red blood cell membranes, a classic mechanism of anti-inflammation, providing strong evidence for membrane-stabilizing effects (Hasan et al., 2024a). Moreover, this study showed that AA reduced formalin-induced paw edema (e.g., by 40%–60% at 20–40 mg/kg) and pain-related behaviors in chicks *in vivo*. Notably, AA was an antagonist to traditional non-steroidal anti-inflammatory drugs (NSAIDs), including celecoxib and ketoprofen. The effects of NSAIDs were diminished when combined with AA. Moreover, molecular docking and dynamics simulations provided structural support for AA’s antagonistic activity on celecoxib and ketoprofen by showing that AA binds to COX-2 (cyclooxygenase-2) with high affinity, similar to celecoxib, and forms a stable complex with COX-2, suggesting a primary mechanism of direct enzyme inhibition (Hasan et al., 2024a).

The anti-inflammatory effects of AA extend beyond COX inhibition and appear to be crucially mediated through the modulation of key transcription pathways. AA has been shown to act as a dual activator of PPARα/γ. In human dermal fibroblasts, AA inhibited UVB-induced expression of matrix metalloproteinase-1 (MMP-1) via inhibition of the MAPK (mitogen-activated protein kinase) pathway, along with its downstream effectors, activator protein-1 (AP-1) and NF-κB. The effects of AA were shown to be PPARα/γ-dependent, as antagonists of the PPARs, GW6471 and BADGE, blocked the inhibitory actions of AA on MMP-1 expression and inflammatory signaling (Jeon et al., 2015). The inhibition of NF-κB was additionally characterized by AA-mediated inhibition of degradation of the NF-κB inhibitory protein IκBα, as well as prevention of nuclear translocation of the p65 subunit.

AA’s potential pharmacological effects on inflammation have been assessed in various disease-specific inflammatory models. In a model of allergic asthma induced by ovalbumin (OVA), AA treatment reduced airway hyper-responsiveness (e.g., by 50%–70% at 10–40 mg/kg), reduced inflammation and the number of inflammatory cells in bronchoalveolar lavage fluid (eosinophils, neutrophils, lymphocytes), and reduced Th2 cytokines (IL-4, IL-5, IL-13), OVA-specific IgE, and nitric oxide (NO). These effects of AA in ameliorating asthma symptoms were attributed to the inhibition of NF-κB in lung tissues (Gao et al., 2016). In a separate model of atopic dermatitis induced by chemical (2,4-dinitrochlorobenzene, DNCB), AA made into a cream and applied topically was found to reduce skin lesions, dermatitis scores, and serum IgE. These beneficial effects were attributed to suppressing the inducible nitric oxide synthase (iNOS)-COX-2 pathway, and reductions in the transcriptional levels of pro-inflammatory cytokines (TNF-α, IL-1β, IL-4, IL-5, IL-6, IL-10), as well as inhibition of NLRP3 (NOD-like receptor protein 3) inflammasome activation in skin tissues (Park et al., 2023). The effectiveness of AA on skin inflammation was also validated in an imiquimod-induced psoriasis model, where AA lowered the psoriasis area severity index (PASI) scores (e.g., by 30%–50% at 40 mg/kg), reduced epidermal thickness, rebalanced the Th17/Treg cell ratio in the spleen, and inhibited serum concentrations of IL-17A, IL-23, TNF-α and IL-1β (Li X. Q. et al., 2021). In addition, in human osteoarthritis chondrocytes, AA inhibited IL-1β-induced inflammation via the p38 MAPK signaling pathway, by decreasing TNF-α, NO, prostaglandin E2 (PGE2) and key enzymes that promote the degradation of the matrix (MMP-1, MMP-3, and MMP-13). Importantly, the PPARγ antagonist GW9662 reversed the anti-inflammatory actions of AA, indicating the involvement of the PPARγ pathway (Kang et al., 2018).

**FIGURE 4:**
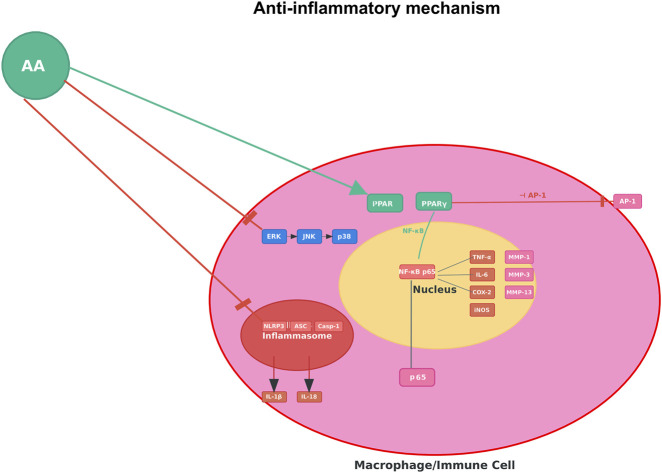
Anti-inflammatory mechanism of AA. Note: Figure 4 illustrates AA suppressing pro-inflammatory signaling (e.g., NF-κB–mediated cascades) in macrophages/immune cells to reduce inflammatory cytokine production.

The body of evidence collectively supports AA as a multi-modal anti-inflammatory agent. AA’s effects may be due to a combination of direct enzymatic inhibition, activation of regulatory nuclear receptors, and downregulation of key pro-inflammatory signaling pathways. This multi-targeted action results in reduced expression and production of a broad array of inflammatory mediators, including pro-inflammatory cytokines, chemokines, NO, PGE2, and MMPs. The consistent efficacy of AA in multiple inflammatory conditions, including inflammatory skin conditions, respiratory conditions, and joint conditions, supports the potential therapeutic application of AA for a variety of clinical indications (Figure 4).

### 3.3 Antimicrobial activity

AA has a broad range of antimicrobial activity, especially against Gram-positive bacteria. For example, AA has been shown to have strong activity against *Staphylococcus aureus*, including methicillin-resistant *Staphylococcus aureus* (MRSA), with an MIC (minimum inhibitory concentration) range of 8–64 μg/mL based on the bacterial strain. Abietic acid’s activity is due to its ability to compromise the bacterial cell membrane. For example, AA was shown to increase uptake of fluorescence dyes such as SYTOX Green in membrane permeability testing, which indicated loss of membrane integrity (de Lima Silva et al., 2023a). Similarly, AA also exerted bacteriostatic activity against *Staphylococcus pseudointermedius*, reducing overall bacterial growth by up to 78% at 6 h in time-kill assays over several concentrations, but was not able to completely kill the bacteria (Buommino et al., 2021). Additionally, AA has activity against Gram-negatives, such as *Pseudomonas aeruginosa*, but at lower levels than corresponding Gram-positive species. The reduced activity is likely due to the structural differences in the cell wall; for example, biofilm viability was reduced by approximately 90% after 30 min of exposure to AA in quantitative biofilm assays (Dziegielewska et al., 2024).

Besides its antibacterial activity, AAis also a noteworthy antifungal agent, particularly in conjunction with a standard antifungal. In terms of *Candida spp*, such as *Candida albicans* and *Candida tropicalis*, AA alone was not significantly fungicidal (MIC > 1024 μg/mL), but was able to enhance fluconazole’s efficacy. Fluconazole’s IC_50_ (half maximal inhibitory concentration) was over 1000 μg/mL alone when used alone, which was reduced to approximately 75–160 μg/mL in combination therapy. Part of the reason for this synergism is that a molecular docking study suggesting that AA interacts with lanosterol 14α-demethylase (CYP51), an important enzyme in ergosterol biosynthetic pathways, and potentiated fluconazole action through competitive inhibition (de Lima Silva et al., 2023a).

AA also inhibited fungal biofilm formation, as well as that of the oral bacterial pathogen *Streptococcus mutans*. This inhibition was measured through crystal violet staining, where biofilm biomass was reduced by approximately 99% at a concentration of 64 μg/mL (Ito et al., 2020). The antibiofilm effects could play an important role for treating persistent infections, since biofilms tend to be resistant to antimicrobials. AA’s antibiofilm properties are not limited to fungi but can also affect biofilms produced by bacteria in various contexts. For example, in ocular infection models, AA encased in bacterial cellulose carriers reduced *S. aureus* and *P. aeruginosa* biofilms on artificial eyeballs by as much as 80%, demonstrating its potential topical application (Dziegielewska et al., 2024). Similarly, with MRSP (methicillin-resistant *Staphylococcus* pseudointermedius), dissolved AA had a significant effect on the viability of preformed biofilms cultivated at sub-MIC doses (i.e., 20 μg/mL). After treatment, viability was reduced by 40%–80%, as determined by XTT assays and confirmed through confocal microscopy, which showed increased red staining implying dead cells embedded in the biofilm matrix (Buommino et al., 2021). These studies reveal that AA can penetrate biofilm matrices and kill organisms of clinical importance, which is a major advantage over many traditional antibiotics that cannot effectively penetrate biofilm resistance.

Although results are encouraging, the antimicrobial effects of AA are variable in terms of both bacterial species and exposure conditions. For example, AA was more effective against *S. aureusthan* it was against *P. aeruginosa*, and it also took longer for AA to affect *C. albicans* biofilms (24 h) when compared to the bacteria biofilms (30 min) (Dziegielewska et al., 2024). Along the same lines, in a number of situations, AA showed a bacteriostatic mode of action rather than that of a bactericidal agent. Specifically, with *S. mutans*, AA was able to inhibit growth and acid production, but without substantially compromising membrane integrity, which suggests that it may be better suited for preventative or adjunctive therapies as opposed to acute treatment (Ito et al., 2020). Notably, AA exhibited low cytotoxicity at the antimicrobial concentrations, up to 256 μg/mL in human fibroblasts and keratinocytes cell lines, which provides some evidence for its potential therapeutic safety. However, higher doses could be cytotoxic in monocytic cells.

**FIGURE 5:**
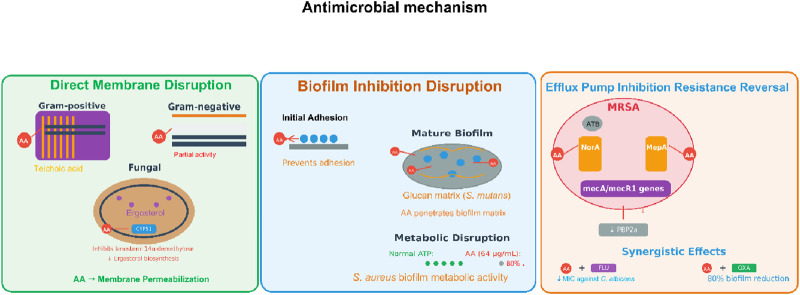
Antimicrobial mechanism of AA. Note: Antimicrobial actions include direct membrane permeabilization (Gram±/fungal), efflux pump inhibition (MRSA resistance reversal), and biofilm disruption (adhesion prevention, matrix penetration, metabolic impairment).

AA can be considered a multi-faceted antimicrobial that could be used to treat bacterial and fungal infections, particularly due to its antibiofilm and synergistic mechanisms; however, more research is required on possible delivery systems and to evaluate efficacy in humans. Considering AA’s natural source and various mechanisms of action, it may be conceptualized as a beneficial addition to tackle antimicrobial resistance, thus research into its pharmacological applications can continue (Figure 5).

**FIGURE 6:**
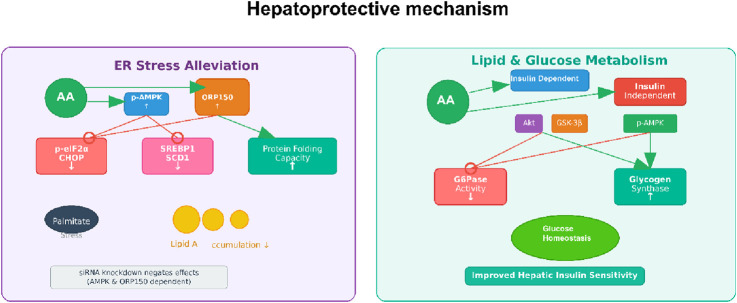
Hepatoprotective mechanism of AA. Note: Figure 6 illustrates AA’s hepatoprotective effects, including ER stress alleviation (by activating p-AMPK and ORP150 to reduce ER stress markers like p-eIF2α/CHOP and SREBP1, and enhance protein folding) and regulation of lipid and glucose metabolism (improving hepatic insulin sensitivity via modulating insulin signaling, GSK-3β, and AMPK to maintain glucose homeostasis).

### 3.4 Hepatoprotective activity

Among the several pharmacological effects of abietic acid, its beneficial hepatoprotective effects have been studied the most in different liver injury models. An et al. demonstrated that AA was protective against acetaminophen (APAP)-induced acute liver injury (An et al., 2023). In their studies, AA greatly reduced the pathological changes in the liver associated with APAP administration, such as necrosis and infiltrating inflammatory cells. There were also significantly lower serum levels of the enzymes ALT and AST (e.g., reduced by 50%–70% at 25–50 mg/kg), which are indicative of hepatic injury. Mechanistically, AA acted to diminish inflammation, represented by decreased production of TNF-α and IL-1β, and by blocking the NF-κB pathway. A unique feature of AA was its marked capacity to inhibit ferroptosis, which is a regulated form of cell death characterized by lipid peroxidation and iron accumulation. Importantly, AA treatment reversed the APAP-induced elevations of MDA (malondialdehyde) and Fe^2+^ while restoring the depletion of GSH (glutathione), ATP (adenosine triphosphate), and the expression of GPX4 and xCT (cystine/glutamate antiporter) expression. The beneficial effects of AA were shown to be dependent on the activation of the Nrf2/HO-1 (nuclear factor erythroid 2-related factor 2/heme oxygenase-1) axis, because the protective effects of AA were eliminated with Nrf2-knockout mice and Nrf2-knockdown hepatocytes. Overall, the evidence supports Nrf2 activation as a significant and beneficial mechanism underlying the hepatoprotective capacity of AA in the liver.

In addition to acute toxicant-induced injury, AA also has the potential to address liver injury associated with metabolic stress. Jung et al. explored the effects of AA on ER (endoplasmic reticulum) stress and lipid accumulation in human primary hepatocytes treated with palmitate, a treatment paradigm that models non-alcoholic fatty liver disease (NAFLD) (Jung et al., 2022). They found that exposure to AA decreased lipid accumulation and the levels of lipogenesis-related proteins such as SREBP1 (sterol regulatory element-binding protein 1) and SCD1 (stearoyl-CoA desaturase 1). AA also reduced ER stress induced by palmitate, as indicated by lower levels of phospho-eIF2α (phosphorylated eukaryotic initiation factor 2 alpha) as well as CHOP (C/EBP homologous protein), and decreased apoptosis. They elegantly showed that these effects were mediated through the AMPK/ORP150 (adenosine monophosphate-activated protein kinase/oxygen-regulated protein 150) signaling pathway. siRNA-mediated knockdown of either AMPK or ORP150 reversed the beneficial effects of AA on ER stress, lipogenesis, and apoptosis, thus confirming the importance of this axis. Overall, these findings suggest that AA can modulate hepatic lipid metabolism and ER homeostasis and may represent a future treatment option for individuals at risk of NAFLD.

The hepatoprotective activity of AA is also evident in models of inflammation. For example, Ramnath et al. found that AA reduced LPS (lipopolysaccharide)-induced liver injury in BALB/c mice (Ramnath et al., 2016). In their study, histopathological analysis showed that AA treatment prevented the lymphocytic infiltration and necrosis associated with LPS. The authors suggested that the protective effect may be attributed to the anti-inflammatory properties of abietic acid, possibly due to its hydroxyl groups, and may involve the reduction of inflammatory mediators such as NO.

AA is a naturally derived compound with diverse functions and substantial hepatoprotective potential against a range of insults, including drug overdoses and inflammation. Its mechanisms of action have common features that activate critical Nrf2/HO-1 pathways for antioxidant and anti-ferroptosis action, AMPK/ORP150 for alleviating ER stress and preventing lipid accumulation, and NF-κB for anti-inflammatory action. These crosstalk pathways underscore AA’s potential as a drug development candidate. It is important to point out that the evidence to date is based primarily on preclinical models. Further research is required to assess its therapeutic potential and advance to clinical models to determine translatability to human liver disease (Figure 5).

### 3.5 Wound healing and tissue repair

Recent research has demonstrated AA’s effectiveness through multiple mechanisms (e.g., promoting angiogenesis), and complex topical delivery systems have been developed to maximize its bioavailability and achieve sustained release at the wound site. A seminal study conducted by Park et al. directly demonstrated the pro-healing effects of AA *in vitro* and *in vivo* (Park et al., 2017). In this study, AA was found to significantly promote both cell migration (e.g., by 2-3 fold at 0.8 μM) and tube formation in human umbilical vein endothelial cells, which are essential steps in angiogenesis. The study linked the angiogenic effects of AA to the activation of key signaling pathways, mediated by upregulating phosphorylated extracellular signal-regulated kinase (p-ERK) and p38 mitogen-activated protein kinase. In addition, in a mouse model of cutaneous wounds, treatment with AA (0.8 µM) showed significantly increased rates of wound closure compared to controls, with accelerated healing noted as early as day two post-treatment. The authors reported that the wound closure effects were likely mediated through stimulating neovascularization, an important part of delivering nutrients and oxygen during tissue regeneration (Park et al., 2017).

Mirgorodskaya et al. addressed the challenge of formulating hydrophobic AA by developing and characterizing biocompatible microemulsions and emulgels as novel drug delivery systems (Mirgorodskaya et al., 2022). An important finding was that the microemulsions and emulgels containing a carbamate-containing surfactant and Carbopol® 940 significantly prolonged AA release. The sustained release profile was consistent with a near-zero order kinetic model, which would be favorable for sustaining effective drug concentrations over long periods of time. Furthermore, the formulations showed high antioxidant activity in tests using chemiluminescence, which may also allow for reduction of oxidative stress in the wound environment. *In vivo* effectiveness was demonstrated in a Wistar rat incision model: rats treated with topically applied AA-loaded formulations (0.5% wt.) showed rapid healing and doubled the tissue tensile strength compared to the untreated control group. The remarkable efficacy was attributed to a drug therapy that utilized the anti-inflammatory effects of AA combined with additional antibacterial effects from formulation components such as oleic acid (Mirgorodskaya et al., 2022).

**FIGURE 7:**
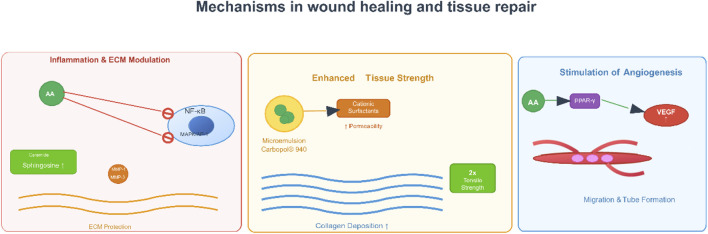
Mechanisms in wound healing of AA. Note: Figure 6 outlines its roles in wound healing and tissue repair, encompassing inflammation and ECM (extracellular matrix) modulation (via NF-κB and MMP regulation for ECM protection), angiogenesis stimulation (through PPAR-γ-mediated VEGF (vascular endothelial growth factor) induction to promote endothelial migration and tube formation), and enhanced tissue strength (via increased collagen deposition and improved tissue toughness).

The wound-healing properties of AA are twofold. First, it exerts direct biological effects by promoting angiogenesis—via activating MAPK signaling pathways—and stimulating the proliferation and migration of cells critical for tissue repair. Second, the incorporation of AA into microemulsions and emulgels improves its therapeutic use through sustained release and better adhesion to the skin, while additionally providing antioxidant and antimicrobial activity. Abietic acid’s natural pharmacological activity combined with the delivery system advantages make a strong argument for further investigating this topical agent in wound care. Future studies should consider clinical studies to demonstrate these promising preclinical findings and examine its potential in combination with other wound-healing agents (Figure 7).

### 3.6 Others

Research has shown that AA may reduce hyperuricemia, pain, emesis, lung injury, osteoporosis, and diabetic nephropathy, primarily through modulation of key signaling pathways and molecular targets.

In hyperuricemia, AA has been shown to have inhibitory effects on xanthine oxidase (XO), which is an important enzyme for uric acid production. Indeed, Dai et al. identified AA from sunflower calathide extract that inhibited XO with an IC_50_ of 10.60 µM and a Ki of 193.65 nM, with no cytotoxicity to HEK293T cells (Dai et al., 2023). Transcriptomics analysis showed AA modulates purine metabolism by suppressing purine nucleoside phosphorylase and ribose-phosphate pyrophosphokinase genes, suggesting there is a mode of action that goes beyond inhibiting XO and may regulate purine biosynthesis. The dual action of AA supports its potential as a natural alternative to conventional XO inhibitors like allopurinol.

AA exhibits substantial analgesic effects. Silva et al. found that administration of AA, at doses of 50, 100 and 200 mg/kg was able to reduce carrageenan-induced paw edema and decrease MPO (myeloperoxidase) levels in mice, which signified inhibition of neutrophil infiltration (de Lima Silva et al., 2023b). In dextran-induced edema models, AA antagonized histamine-dependent pathways, similar to promethazine, as well as partially inhibited eicosanoid-mediated responses. The compound was able to reduce nociception in formalin and acetic acid-induced writhing tests, indicating involvement of both neurogenic and inflammatory pain pathways, but none of these effects occurred with CNS depression. AA seems to provide improved safety compared to current NSAIDs considering the anti-inflammatory and analgesic effects.

Additionally, the antiemetic effect of AA has been evaluated in chick models where it was found to increase latency and reduce retching induced by copper sulfate. Hasan et al. found that both 5HT3 (5-hydroxytryptamine receptor 3) and muscarinic receptors were involved and supported this finding with molecular docking as AA had the highest binding affinity to M4 (−10.2 kcal/mol) and 5HT3 (−8.1 kcal/mol) compared to the reference drug ondansetron (Hasan et al., 2024a). This variation in receptor binding could help explain how it can synergize with other standard antiemetics for management of nausea and vomiting.

In lung injury models, AA possesses protective effects by regulating immune responses. Fang et al. noted that AA reduced sepsis-induced lung injury in mice by inhibiting activation of the NF-κB pathway, reducing the polarization of M1 macrophages, and promoting the shift towards M2 phenotype and reduced pro-inflammatory cytokines (IL-1β, TNF-α, IL-6) (Fang et al., 2022). Likewise, Li et al. observed that AA restored the balance between Th17/Treg in an acute lung injury model via PPARγ activation while attenuating oxidative stress and inflammation (Li et al., 2022). These studies suggest that AA treatment may be beneficial in lung inflammatory diseases.

The osteoprotective effects of AA have been examined in osteoporosis models. Thummuri et al. showed that AA inhibited RANKL (receptor activator of nuclear factor kappa-B ligand)-induced osteoclastogenesis in RAW264.7 cells by inhibiting NF-κB and MAPK signaling, leading to reduced expression of osteoclast markers (TRAP (tartrate-resistant acid phosphatase), NFATc1 (nuclear factor of activated T-cells cytoplasmic 1)) (Thummuri et al., 2018). Furthermore, *in vivo* experiments revealed that AA reduced LPS-induced bone loss by reducing serum levels of TNF-α and IL-6, further supporting the utility of AA in treating osteolytic diseases.

One of the most promising areas of application might be diabetic nephropathy where AA can alleviate kidney injury. The authors reported that in diabetic rats induced by a high-fat diet and streptozotocin, AA (10–20 mg/kg) exerted beneficial effects by decreasing oxidative stress via stimulating the Nrf2 pathway, increasing antioxidants (SOD (superoxide dismutase), CAT (catalase), GPx (glutathione peroxidase)), and decreasing RAGE (receptor for advanced glycation endproducts) and Keap1 (Kelch-like ECH-associated protein 1) levels. This significant reduction in oxidative stress is likely a result of reduced inflammation (NF-κB, IL-6, TNF-α), fibrosis (TGF-β1 (transforming growth factor beta 1), GSK-3β (glycogen synthase kinase 3 beta)), and apoptosis (caspase-3, Bax) and improvement in parameters of renal function (Wahab et al., 2022) (Table 1).

**TABLE 1:** The main pharmacological properties of AA.

Pharmacological activities	Key findings	Doses/IC_50_	Study models	*In vitro*/*In vivo*	References
Anti-tumor	Induced ferroptosis via HO-1 pathway; synergistic with chemotherapeutics	10–20 μM (IC_50_ *in vitro*); 10–20 mg/kg (*in vivo*)	T24 and 5637 cells, nude mice	Both	Xu et al. (2023)
	Inhibited migration/invasion via PI3K/AKT/mTOR pathway	20 μM (IC_50_)	HNE1 cells	*In Vitro*	Wen et al. (2019)
	Inhibited IKKβ/NF-κB signaling	10–20 μM (IC_50_)	NSCLC cell lines	*In Vitro*	Liu et al. (2019)
	Reduced lung metastasis via MMPs and Akt inhibition	50 μM (*in vitro*); 20–40 mg/kg (*in vivo*)	B16F10 cells, xenografted mice	Both	Hsieh et al. (2015)
	Reduced MMPs and CKs expression in UVC/DMBA mice	0.5% w/w cream	UVC/DMBA-induced skin cancer mice	*In Vivo*	Yang and Tian (2017)
Anti-inflammatory	Inhibited COX-2; reduced paw edema and cytokine levels	20–40 mg/kg (*in vivo*); 15.69–27.53 μg/mL (*in vitro*)	Chick paw edema model, RAW264.7 macrophages	Both	Hasan et al. (2024b)
	Reduced inflammation, restored skin structure; scavenged free radicals	0.5% w/w cream	DNCB-treated BALB/c mice	*In Vivo*	Park et al. (2023)
	Reduced IL-1β-induced inflammation via PPARγ activation	1–10 μM	Human osteoarthritis chondrocytes	*In Vitro*	Kang et al. (2018)
	Reduced allergic airway inflammation via NF-κB inhibition	10–40 mg/kg	OVA-induced asthma mice	*In Vivo*	Gao et al. (2016)
	Inhibited UVB-induced MMP-1 via PPARα/γ activation	1–10 μM	Hs68 human dermal fibroblasts	*In Vitro*	Jeon et al. (2015)
	Reduced Th17/Treg imbalance and modulated gut microbiota	40 mg/kg	IMQ-induced psoriasis mice	*In Vivo*	Li X. Q.et al. (2021)
Antimicrobial	Effective against *S. aureus*, *P. aeruginosa*, and C. albicans biofilms; reduced lethality in Galleria mellonella	256–512 μg/mL	Artificial eye models, G. mellonella larvae	Both	Dziegielewska et al. (2024)
	Enhanced fluconazole activity against C. albicans and *C. tropicalis*	1065–3255 μg/mL (IC_50_)	*Candida* spp.	*In Vitro*	de Lima Silva et al. (2023a)
	Synergized with oxacillin against methicillin-resistant S. pseudintermedius	32–64 μg/mL	*S. pseudintermedius*	*In Vitro*	Buommino et al. (2021)
	Inhibited S. mutans growth and biofilm formation	0.1–1.0 mg/mL	*S. mutans*	*In Vitro*	Ito et al. (2020)
Hepatoprotective	Reduced inflammation and ferroptosis via Nrf2/HO-1 axis	25–50 mg/kg	APAP-treated mice	*In Vivo*	An et al. (2023)
	Reduced lipid accumulation via AMPK/ORP150 signaling	10–20 μM	Human primary hepatocytes	*In Vitro*	Jung et al. (2022)
	Attenuated LPS-induced liver injury in mice	10–20 mg/kg	LPS-treated BALB/c mice	*In Vivo*	Ramnath et al. (2016)
	Regulated G6Pase and GS via AMPK/Akt pathways	1–10 μM	Hepatocytes, diabetic mice	Both	Nachar et al. (2015)
Wound healing	Accelerated incised wound closure in rats; antibacterial/anti-inflammatory	0.5% w/w	Wistar rats	*In Vivo*	Mirgorodskaya et al. (2022)
	Enhanced angiogenesis via ERK/p38 MAPK in HUVECs	0.8 μM; 0.5% w/w cream	HUVECs, cutaneous wound mice	Both	Park et al. (2017)
Renal protection	Improved cell viability in hypertonic-stressed MDCK cells	4.31 μg/mL	MDCK cells	*In Vitro*	Li S. et al. (2021)
	Reduced oxidative stress, inflammation, and fibrosis in diabetic rats	10–20 mg/kg	High-fat diet/STZ-induced diabetic rats	*In Vivo*	Wahab et al. (2022)
Osteoprotective	Inhibited osteoclastogenesis via NF-κB/MAPK pathways	1–10 μM; 20–40 mg/kg	RANKL-induced osteoclasts, LPS-induced osteolysis mice	Both	Thummuri et al. (2018)
Antiedematogenic/Analgesic	Reduced paw edema; inhibited myeloperoxidase and histamine	50–200 mg/kg	Carrageenan/dextran-induced edema models	*In Vivo*	de Lima Silva, et al. (2023b)
Anti-hyperuricemia	Inhibited xanthine oxidase (IC50 = 10.60 μM); regulated purine metabolism	10–20 μM	HEK293T cells	*In Vitro*	Dai et al. (2023)
Skin injury protection	Improved UVC-induced skin damage via lipid/purine metabolism pathways	0.5% w/w cream	UVC-irradiated mice	*In Vivo*	Yang and Tian (2017)
Lung injury protection	Restored Th17/Treg balance via PPARγ activation	10–20 mg/kg	LPS-induced ALI mice	*In Vivo*	Li et al. (2022)
	Inhibited M1 macrophage polarization via NF-κB pathway	20–40 mg/kg	Cecal ligation/puncture mice	*In Vivo*	Fang et al. (2022)
Antiemetic	Increased emetic latency and reduced retching frequency; interacted with 5HT3 and muscarinic receptors	20–40 mg/kg	2-day-old chicks treated with CuSO4	*In Vivo*	Hasan et al. (2024a)

## 4 Pharmacokinetics

The pharmacokinetic characteristics of AA, while partially characterized, have new evidence suggesting significant challenges for AA in terms of absorption, distribution, and elimination. An important aspect of AA metabolism is the formation of glucuronide conjugates that are excreted into bile, as is the case for glycyrrhetinic acid, which is eliminated via biliary excretion as a glucuronide conjugate (Lee and Kim, 2019). The biliary excretion of AA glucuronide is presumably mediated by the glucuronide efflux transporter MRP2 (multidrug resistance-associated protein 2), suggesting active transport as a potential pathway for elimination (Svoboda et al., 1999).

The oral bioavailability of AA in mammals has not been determined, but there is indirect evidence that it is low. Oleanolic acid, a structural analogue of AA, exhibited an oral bioavailability of < 5% in rats, which may indicate AA is subject to similar absorption limitations in the intestine (Shang et al., 2021). In addition, this finding is supported by *in vitro* permeability studies using Caco-2 cell monolayers; AA gave an apparent permeability coefficient of 0.41 ± 0.03 ×10^−6^ cm/s, suggesting that it possesses low permeability (Shirai et al., 2022). The low permeability is likely due to the molecular polarity of AA and potential interactions with intestinal efflux transporters.

The limited aqueous solubility of AA due to its rigid tricyclic diterpenoid structure (with conjugated double bonds and a carboxylic acid group) greatly limits its dissolution rate in gastrointestinal fluids, which affects the absorption through the oral route. To deal with these limitations, novel drug delivery approaches are being investigated. For example, in preclinical models, the successful development of AA-loaded hybrid polymeric nanoparticles (AAHNPs) has been reported. The AAHNPs allow for improved solubility of AA, protecting it from degradation in gastrointestinal conditions, and reducing barriers to systemic absorption, potentially increasing anti-inflammatory and antioxidant activity (Han et al., 2024; Han et al., 2025; Zafar et al., 2024).

With respect to distribution, it is likely that AA does not penetrate well into the central nervous system. Measured brain-to-plasma ratios (<0.25) are low, consistent with the inability of other similar lipophilic acids to cross the blood-brain barrier (Lee and Kim, 2019). Furthermore, pharmacokinetic modeling with the analogous glycyrrhetinic acid suggests that AA accumulates in the liver, which likely reflects a common pharmacokinetic property of compounds undergoing considerable biliary excretion.

Understanding AA’s pharmacokinetics requires careful consideration of enterohepatic recycling. Glucuronide metabolites in the bile can be converted back to the parent aglycone by the action of bacterial β-glucuronidase in the intestinal lumen, which allows for the reabsorption of AA into the bloodstream. This phenomenon potentially explains the double-peak phenomenon of the AA plasma concentration-time profile for AA and prolonged systemic exposure, both of which impact potency and toxicity. Therefore, it will be important for future studies of the pharmacokinetics of AA to separate early (0–24 h) and late (>24 h) fecal samples to investigate recycling of AA and account for the unabsorbed drug.

The existing knowledge of AA’s pharmacokinetics is mainly based on extrapolation from other structural analogues, and *in vitro* systems. The recognition and documentation of glucuronidation and biliary excretion of AA highlight the contribution of transporters and gut microbiota to overall disposition. Due to the recognized solubility and permeability challenges, there is a sustained interest in newer and innovative delivery systems like nanoparticle encapsulation. Given the consideration of enterohepatic recycling, AA’s pharmacokinetic profile would greatly benefit from quantitative *in vivo* pharmacokinetic data, including absolute bioavailability, apparent volume of distribution, and clearance (Table 2).

**TABLE 2:** Key pharmacokinetic parameters and challenges of AA.

Parameter	Characteristic/Value	Implication
Oral bioavailability	Not quantitatively determined; expected to be low	Limited systemic exposure; necessitates enhanced formulation strategies
Caco-2 permeability	0.41 ± 0.03 × 10^−60^.41 ± 0.03 × 10^−6^ cm/s	Classified as a low-permeability compound; weak passive diffusion
Biliary excretion	Significant; mediated via glucuronidation and MRP2 transport	Major elimination route; potential for enterohepatic circulation
CNS distribution	Brain-to-plasma ratio < 0.25	Restricted penetration across the blood-brain barrier
Primary distribution site	Likely hepatic accumulation	Aligns with biliary elimination pathway; may have implications for liver effects

The studies in section 3 show that although AA does not readily penetrate and be absorbed into the body, AA does reach systemic concentrations in certain types of lung injury models with high enough levels to be tested as treatment at an anti-metastatic model. The majority of animal studies utilizing AA show average decreases in lung metastases when testing dosages of AA between 20 and 40 mg/kg. There are a number of reasons that these studies could yield the results they do, including: enterohepatic recirculation allowing the gradual accumulation of small (µg/kg) doses of AA over time yields therapeutic effects to the subject; the effects of AA on the local tissues (i.e., secretions of enzymes, making them more susceptible to being damaged from the agent); possible differences between species of subjects used, including that the potential differences in absorption of AA determined from experimental studies of one species may not apply to other species. An additional concern related to potential limitations associated with human use and testing of the doses utilized in these studies should be noted; that is to say, using such high doses in the animal models to evaluate the therapeutic effects of AA may negate the potential limitations related to the limited bioavailability of AA. Based on the evidence above, it is imperative that direct PK-PD studies be conducted to confirm the mechanisms by which the therapeutic effects are achieved, and to enhance the ability to optimize dosing for patient treatment of AA. In the absence of such evidence regarding PK-PD relationships for AA, the potential for a future human clinical trial utilizing AA remains uncertain.

## 5 Toxicological profiles

AA is generally considered to have a fairly acceptable safety profile, in line with its origin from natural products, specifically coniferous resins. Nonetheless, complete toxicology studies are necessary to determine its therapeutic window for development as a pharmaceutical agent.

There is a limited amount of acute toxicity data for AA in mammalian models, and there are no reported median LD_50_ values from studies. Regulatory summaries and repeat-dose studies suggest low acute oral toxicity; for example, there were no adverse events in mice receiving 250 mg/kg/day for 28 days or in rats treated with 100 mg/kg/day for an estimated 15 weeks, indicating a high degree of interspecies compatibility at these doses (Bampidis et al., 2023). A recent *in silico* toxicity prediction estimated an oral LD_50_ of 715 mg/kg in rats, with no significant risk for hepatotoxicity, carcinogenicity, or mutagenicity (Hasan et al., 2024b). The model also suggested a potential immunotoxic concern; however, this remains a hypothetical risk generated by computational methods and requires experimental validation. Priority should be given to the available *in vivo* data, which do not indicate acute toxicity at tested doses, while acknowledging that *in silico* models provide preliminary insights with varying confidence levels depending on the algorithm and training data.

On a molecular scale, AA shows an interesting paradoxical redox behavior as an antioxidant and a pro-oxidant depending on concentration and cellular microenvironment. In pancreatic and breast cancer cell lines (MCF-7), AA can drive selective cytotoxicity, including inducing oxidative stress responses (Haffez et al., 2022). The concentration specific to nrf2/hO-1 is shown in studies where low doses (1–10 μM) will activate Nrf2/H0-1 pathways causing antioxidant-driven liver model; at higher concentrations (20–50 μM), it causes Pro-oxidant Cytotoxicity by creating excessive levels of ROS while downregulating gpX4 and causing Feralptosis within cancer cells. The significance of establishing a dose range for therapeutic utilization is emphasized due to preclinical data showing antioxidants providing benefit to normal cells at concentrations less than 20 μM while exhibiting pro-oxidant activity above this number with malignant cells and cells that are under stress, thus creating the ability to selectively target those neoplastic cells. It must be noted that the defined range varies based on cell type and exposure time; therefore, a necessary step in the process of defining an effective method of therapy is conducting multiple dose-response studies to prevent off-target oxidative injury in non-cancerous tissues during application of the therapeutic.

While the available information from animal toxicological studies suggests that AA does not pose a serious risk, for a full risk assessment we would require more solid and systematic *in vivo* toxicological studies. There is currently no defined LD_50_, and the body of evidence includes only a very limited number of repeated-dose studies. In addition, the predicted immunotoxicity from *in silico* studies represents a preliminary flag that warrants targeted experimental follow-up, such as immune cell assays, but it should not overshadow the reassuring *in vivo* tolerance data (Ahmad et al., 2024; Feitosa et al., 2025). The concentration-dependent dual redox activity of AA also raises questions about how much to dose in vivo therapeutic applications, in order to avoid oxidative damage to non-target tissues. Importantly, future studies should focus upon identifying the no-observed-adverse-effect-level (NOAEL), the mechanisms behind the immunotoxic potential, and the chronic long-term toxicity of AA (Table 3).

**TABLE 3:** Summary of preclinical toxicological findings for AA.

Toxicity endpoint	Findings	Interpretation and implications
Acute oral toxicity	No adverse effects in mice (250 mg/kg/day, 28 days) and rats (∼100 mg/kg/day, 15 weeks)	Suggests low acute toxicity and good tolerance at these repeated doses
Computational prediction	Predicted LD_50_: 715 mg/kg (rat); No hepatotoxicity/carcinogenicity/mutagenicity; Potential immunotoxicity	Provides preliminary safety insights; immunotoxicity prediction needs verification
Redox activity	Pro-oxidant (cytotoxicity in cancer cells) and antioxidant (at lower doses) effects observed	Indicates a dual nature; therapeutic application must consider dose and context

## 6 Conclusion

AA is a pharmacologically diverse natural product with the potential to treat many clinical conditions, including cancer, inflammatory conditions, infections, and metabolic diseases. AA has been studied extensively in preclinical models, and it has a favorable toxicological profile, which supports further development. Nonetheless, a successful transition from AA to clinical medicine will require a collaborative interdisciplinary effort between medicinal chemistry, pharmaceutical sciences, molecular pharmacology and clinical medicine to fill the gaps in our knowledge about pharmacokinetics, mechanisms of action, and long-term safety of these compounds. Identification of optimized derivatives or more sophisticated formulations, combined with evidence-based evaluations will be a prerequisite to successful development of therapeutics based on this historic natural product. The recognition that natural product scaffold systems are important for modern drug discovery continues to expand in the pharmaceutical community, and AA represents both the potential and challenges of advancing therapies originally derived from either traditional knowledge. By providing sustained funding for research and support to develop these therapies, AA or its derivatives will hopefully become part of the therapeutics available for previously untreatable conditions while honoring the traditional knowledge of the medicinal value of coniferous resins using the standards of modern pharmaceutical science.

## 7 Further perspectives

The narrative literature review has limitations which must be noted and considered. To start with, most literature was based on preclinical research, predominately *in vitro* and animal model studies. As a result of the differences in species, the clinical application of these studies will be limited because of the differences in metabolism and response between species to various drugs, making it difficult to extrapolate the data to humans. In addition, the studies included in this review had varying degrees of quality and were typically small studies. As a result of this lack of consistency in study size or study design, there are differences in the quality of the data being evaluated and, thus, the estimated effects may be overstated because of the lack of standardization in methodology. Finally, there are significant gaps in the available literature, including a lack of long-term toxicology studies, a lack of clinical pharmacokinetic studies, and a lack of understanding of the overall mechanisms involved in different disease models. These limitations highlight the need for more thorough and systematic reviews and clinical trials to corroborate these findings.

Therefore, it is important to recognize and address several critical gaps in the current knowledge base to move AA towards clinical practice. First, the overall absence of quantitative *in vivo* pharmacokinetic data in mammals, as well as several important metrics that typically include absolute bioavailability, volume of distribution, clearance, and enterohepatic recycling on systemic exposure, is a limitation that should not be ignored (Elias and Konig, 2025; Zhang et al., 2025). Pharmacokinetic studies with validated analytical methods are fundamental to establish dose-response relationships with observed effects, as well as predict human pharmacokinetics. Second, although preclinical efficacy has been described in some disease models, the lack of purposefully designed clinical trials disallows one to be certain of therapeutic efficacy and safety in humans. Phase I trials will be necessary to ascertain the safety profile, maximum tolerated dose, and pharmacokinetic properties in healthy volunteers followed by Phase II trials designed to assess efficacy of therapy in specific disease indications (Kp Jayatunga et al., 2024). Third, the molecular mechanisms underpinning some of AA’s pharmacological efficacy remain partially characterized. Briefly, while AA has been shown to induce ferroptosis and activate PPARγ, the specific molecular interactions, downstream signaling cascades, and implications of off-target effects remain to be identified and studied further using more advanced molecular biology techniques including proteomics, transcriptomics, and CRISPR-based genetic screens (Naqvi et al., 2025). Fourth, the potential for immunotoxicity predicted from *in silico* modeling experiments needs to be fully validated through rigorous immunotoxicity testing, such as assessment of immune cell populations, cytokine production, and hypersensitivity (Lebre et al., 2022). Fifth, there are no long-term toxicity studies (e.g., chronic exposure and reproductive toxicology studies), which are needed to establish the no-observed-adverse-effect-level (NOAEL) and establish safety margins for chronic therapeutic use (Beckett et al., 2023).
